# Mental health professionals’ views and experiences of antipsychotic reduction and discontinuation

**DOI:** 10.1371/journal.pone.0218711

**Published:** 2019-06-20

**Authors:** Ruth E. Cooper, Éanna Hanratty, Nicola Morant, Joanna Moncrieff

**Affiliations:** 1 Newham Centre for Mental Health, Unit for Social and Community Psychiatry, Queen Mary University of London, London, United Kingdom; 2 Research & Development Department, Goodmayes Hospital, North East London NHS Foundation Trust, Essex, United Kingdom; 3 Division of Psychiatry, University College London, London, United Kingdom; University of Toronto, CANADA

## Abstract

**Background:**

The widely established treatment for psychosis is long-term antipsychotic medication. However, many people stop taking this treatment, and request other options. There are also growing concerns about adverse effects, but currently no professional guidelines to support reducing or stopping these drugs. The views and experiences of individual mental health professionals around reducing and stopping antipsychotics are therefore crucial in treatment decisions.

**Methods:**

We conducted 7 focus groups with prescribing psychiatrists and other members of community-based statutory mental health services in London. Participants discussed their views about, experiences, and processes of antipsychotic reduction and discontinuation. Data were analysed using thematic analysis.

**Results:**

Participants acknowledged that antipsychotics can have severe adverse effects. They were generally supportive of trying to reduce these drugs to the lowest effective dose, although stopping antipsychotics was less acceptable. Prior experiences of adverse events after reduction or discontinuation meant that both were approached with caution. Reduction was also reported to be hampered by organisational and knowledge barriers. Lack of resources, pressure to discharge, and poor continuity of care were seen as organisational barriers. Knowledge barriers included inadequate evidence about who might be best suited to reduction, and lack of guidance about how this could be done safely. This meant that reduction was often prompted by patients, and sometimes actively discouraged, and stability with maintenance treatment was often favoured.

**Conclusions:**

Concerns about risk and other barriers means that clinicians are often reluctant to implement reduction or discontinuation of antipsychotic medication. In order to increase the treatment options available to service users, more research and guidance on how to minimise the risks of antipsychotic reduction and discontinuation is required to enable clinicians to engage more constructively with service users requests, offering people more choice and control in managing their mental health condition.

## Introduction

Antipsychotics are the primary treatment for people who experience psychosis and are prescribed to nearly all service users [1]. They provide relief for many people and can be effective in alleviating the symptoms of acute psychosis, and preventing relapse [2–4]. Therefore, for people with multiple episode psychosis, continuous, long-term treatment is routine practice [5,6].

However, many people report unpleasant and debilitating effects from antipsychotics [7]. There are also increasing concerns about the serious adverse effects of these drugs, especially when taken on a long-term basis [8,9], which has led to calls for a review of current recommendations on long-term antipsychotic prescription [8]. Concerns include cardiovascular problems [10], weight gain, diabetes [11,12], brain volume reduction [13], tardive dyskinesia, and sexual dysfunction [14,15]. Alongside this up to a third of service users do not respond to these drugs [16]. As a result, many people stop taking antipsychotics of their own accord [17,18], do not take them as prescribed, or would like more choice about how much medication they take and for how long [19].

Research has mainly focused on outcomes following abrupt antipsychotic discontinuation compared with maintenance treatment, finding a greater risk of relapse for those who discontinue, although also showing that not everyone experiences a relapse [20–22]. Few studies have weighed up all the pros and cons of a *gradual* process of antipsychotic reduction and discontinuation. One such trial compared gradual antipsychotic reduction and discontinuation with maintenance treatment. Results showed a higher risk of relapse in the reduction group in the short term, but in the long term there were no differences in relapse or symptoms and the reduction group had greater rates of social recovery [23,24]. Despite these results, there are currently no professional guidelines for reducing antipsychotics which would help to balance the concerns of clinicians with the preferences of service users. This means that the extent to which individual mental health professionals consider antipsychotic reduction and discontinuation, and their attitudes towards this will play a pivotal role in treatment decisions in clinical practice and at the policy level.

Shared decision making is crucial to patient-centred care, and encouraged in the NICE guidelines [25]. Existing research suggests that service users do not feel involved in decisions around antipsychotic prescription, often feel coerced, and would like to have more choice over their treatment [26–28]. In line with this, in a survey of psychiatrists, only 51% reported regularly using shared decision making with people with schizophrenia [29]. There is also some evidence of reluctance to reduce antipsychotics. In outpatient consultations, psychiatrists were less likely to suggest reduction or discontinuation than service users were to request it [30]. Practitioners in nursing homes felt that antipsychotic reduction could lead to a better quality of life, but were concerned about risk and potential relapse [31,32]. However there is little research directly focusing on the views of mental health professionals in secondary care on antipsychotic reduction and discontinuation (e.g. in Community Recovery Teams), where the majority of long-term antipsychotic prescribing is initiated for people with psychotic conditions. Due to this, as part of preparation for a randomised controlled trial of antipsychotic reduction, we asked mental health professionals in secondary care about their attitudes towards, experiences of and the processes around antipsychotic reduction and discontinuation.

## Materials and methods

The study is reported according to the COREQ checklist (Consolidated Criteria for Reporting Qualitative Research) and takes into account guidance from the Hannes criteria for critical appraisal of qualitative research [33,34]. The study was approved by the East of Scotland Research Ethics Service (Ref. 5/ES/0163). Qualitative data were collected via focus groups, allowing us to explore areas and levels of consensus or divergence of opinion that emerged in discussions within groups of mental health professionals.

### Study design, research team, and reflexivity

The study operated within a critical realist perspective, in that participants’ comments were assumed to reflect an underlying reality that is mediated by perceptions, beliefs and social processes, and consideration is given to how broader cultural processes shape knowledge and practice, both in the clinical field of study, and in the research processes we undertook [35,36]. The study was part of the ‘RADAR’ programme (Research into Antipsychotic Discontinuation and Reduction, National Institute for Health Research (NIHR) Programme Grant, see: https://www.ucl.ac.uk/psychiatry/antipsychotic-discontinuation-and-reduction). It was conducted as preparation for a clinical trial investigating whether reducing and potentially stopping antipsychotics with the support of prescribers improves social functioning compared with maintenance treatment for people with schizophrenia spectrum disorders. All authors are involved in the RADAR study and are academic researchers, with backgrounds in psychology, psychiatry and social science. JM is also a consultant psychiatrist and prescriber of antipsychotics. We aimed to work reflexively in acknowledging the epistemological and personal positions that we brought to the research process and our interpretations of the data.

### Setting and participants

Data were collected in mental health clinics in two London-based NHS Mental Health Trusts. These trusts serve socio-demographically mixed populations that include pockets of high deprivation and ethnic diversity in both urban and suburban areas. They provide inpatient and community-based outpatient mental health services for people with a range of diagnoses including psychosis, bipolar disorder, personality disorder, depression, and anxiety. These mental health services are multidisciplinary including psychiatrists, mental health nurses, social workers, support workers, and psychologists. In the community, psychiatrists will often have 4–6 monthly consultations with patients where medication will be discussed. Other members of the team such as nurses or social workers may see patients more regularly if necessary, providing emotional and more practical support, such as help with housing or employment.

Seven focus groups were conducted between July and October 2016 with each group consisting of 3–8 participants. Participants were recruited from community-based mental health services and consisted of prescribing and non-prescribing mental health practitioners who were psychiatrists, mental health nurses, social workers or clinical team managers. All participants had experience of working with people with psychosis. We recruited from mental health teams that we aimed to involve in the RADAR antipsychotic reduction trial. Participants were invited to participate in the focus groups via email invitations and groups were conducted with all who took up the invitation. It was not possible to ascertain who declined participation, as some people did not reply to these requests which may have been due to time constraints or missing the email rather than actively declining to take part.

### Data collection

A topic guide was developed which explored four main areas (see S1 Appendix for the full topic guide):

- Experiences and views of reducing and discontinuing antipsychotics.
- The process of antipsychotic reduction and discontinuation, including perceived facilitators and barriers, and service user suitability.
- Detection and definition of relapse.
- Forms and sources of support for antipsychotic reduction.

Groups were facilitated by REC & ÉH and their involvement in RADAR was known to participants. Some participants were also known to the researchers due to participation in other research projects or clinical work. Focus groups consisted of participants and facilitators only. Where possible, separate groups were conducted for psychiatrists and other clinical team members, in order to facilitate open discussion from the perspectives of prescribing and non-prescribing practitioners. Participants were also asked to complete a short demographics questionnaire. Written informed consent was obtained from all participants before data collection, and discussions were audio-recorded, transcribed and anonymised. Each group lasted approximately 60 minutes. Field notes were made by facilitators during the focus groups.

### Data analysis

Data were analysed using thematic analysis with NVivo software (Version 11) [37] by the researchers who facilitated the focus groups (REC & ÉH). In order to enhance validity, regular meetings were held with the two other authors (NM & JM), in which emerging codes, themes and patterns of similarity and difference across the data were critically discussed. The first three transcripts were independently coded by each researcher in order to identify key themes. These were then combined and compared to develop an initial coding framework, which was then used to code further transcripts. Codes were progressively refined and organised into emerging themes and sub-themes, and analysis continued until a coherent account of the variations in the data were met. Reflections and field notes from the researchers who facilitated the focus groups were part of the collaborative analysis process, and informed our understandings of the data. We were aware of the busy working lives of our participants, so did not attempt to do member checking or obtain feedback on our interpretations from participants.

## Results

Seven focus groups were conducted between July and October 2016. They included a total of 35 participants. The composition of each focus group is shown in Table 1. Most participants worked in adult mental health services, with a small number from learning disability services, and one who worked in older adult services. Participant demographics are shown in Table 2. The majority of participants were consultant psychiatrists (n = 18) and psychiatric nurses (n = 13). Each group consisted of between 3–8 participants and lasted approximately 60 minutes.

**Table 1:** Focus group composition.

Focus group	N	Profession	Service (N)
1	8	8 Psychiatric Nurses	Home Treatment Team (HTT) (1), Community Recovery Team (CRT) (3), Access and Assessment Team (AAT) (1), Older Adults (1), Early Intervention Team (EI) (2)
2	4	4 Consultant Psychiatrists	Access and Assessment Team (1), Learning Disability (3)
3	4	4 Consultant Psychiatrists	Community Recovery Team (2), Early Intervention Team (1), Assessment and Brief Treatment Team (1)
4	3	3 Consultant Psychiatrists	Early Intervention Team (1), Community Recovery Team (2)
5	6	3 Consultant Psychiatrists, 1 Specialty Doctor, 1 Manager, 1 Clinical Lead	Community Recovery Team (6)
6	6	5 Nurses, 1 Social Worker	Access, Assessment and Brief Intervention Team (6)
7	4	4 Consultant Psychiatrists	Community Recovery Team & Early Intervention Team (2), Access and Assessment Team (1), Learning Disability (1)

Note. N = Number of participants

**Table 2:** Characteristics of study participants.

Characteristics	
**Gender, N (%)**	
Female	22 (63%)
Male	13 (37%)
**Age, mean (SD)**	43 (8.3)
**Ethnicity, N (%)**	
White British	12 (34%)
African	10 (28%)
White Other	5 (14%)
Bangladeshi	2 (6%)
Indian	2 (6%)
Black Other	1 (3%)
Not given	3 (9%)
**Profession, N (%)**	
Consultant Psychiatrists	18 (52%)
Speciality Doctor	1 (3%)
Mental Health Nurse	13 (37%)
Social workers	2 (6%)
Community Recovery Service Manager	1 (3%)

**Fig 1:**
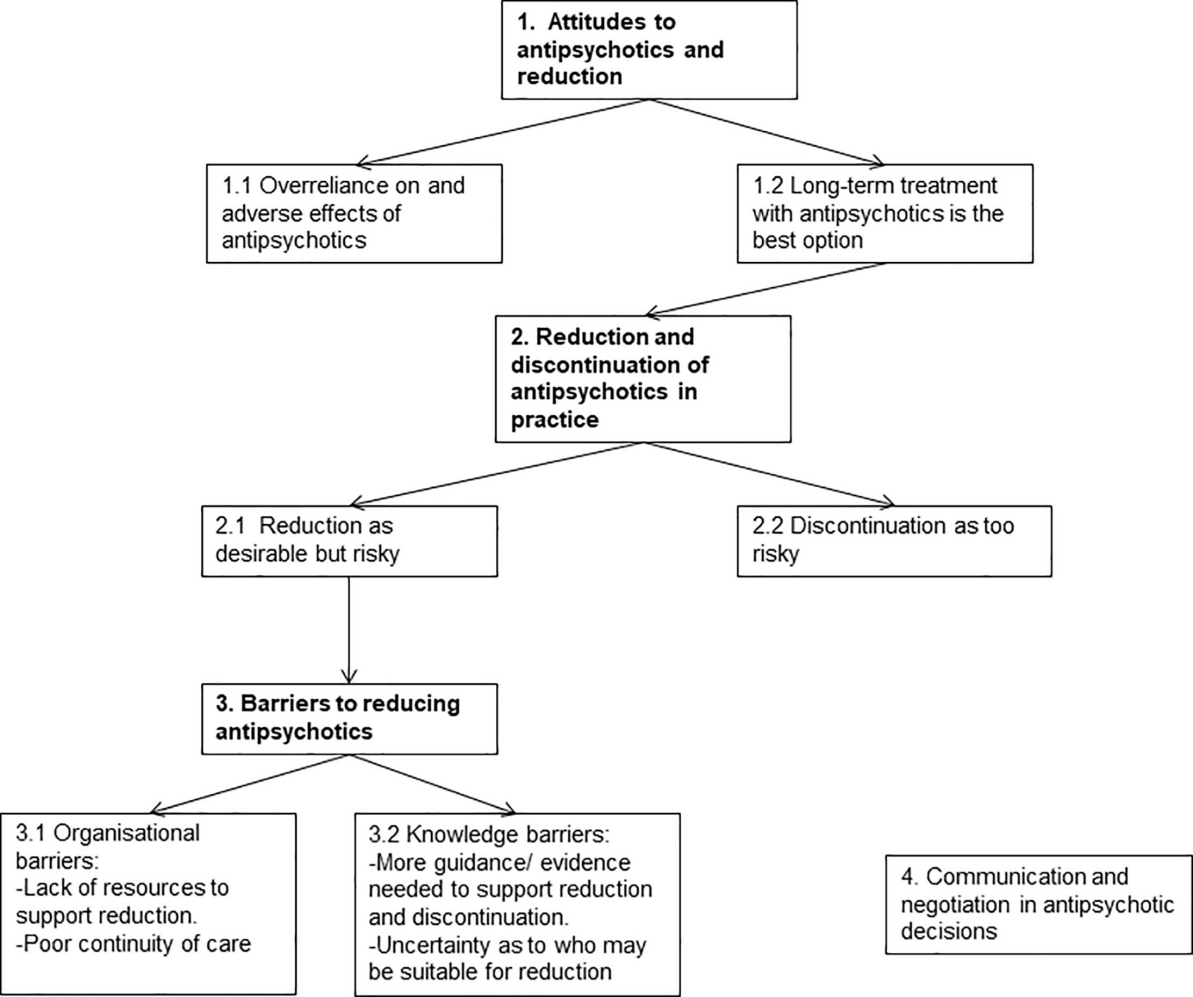
Overview of the developed themes about views and experiences of mental health professionals on reducing and stopping antipsychotics (numbers correspond to sub-sections presented in the results).

Themes that emerged from our analysis, how they relate to each other, and their presentation in sub-sections below are summarised in Fig 1. These encompass practitioners’ views of the role of antipsychotics; their clinical experiences of antipsychotic reduction and discontinuation; their views about organisational and knowledge-based barriers to reducing or stopping antipsychotic treatment; and how decisions about antipsychotics are made and negotiated with service users and families.

### 1. Attitudes to antipsychotics and antipsychotic reduction

In all the focus group discussions, participants acknowledged that antipsychotics can have severe adverse effects, and some criticised what they viewed as overreliance on medication in services. Only a minority of psychiatrists had a completely positive attitude towards long-term antipsychotic treatment, although everyone acknowledged that they had some benefits.

#### 1.1 Over-reliance on and adverse effects of antipsychotics

Across all groups, the serious adverse effects of antipsychotics were acknowledged. Effects that were discussed included weight gain, diabetes, heart problems, sedation, impaired function, sexual dysfunction, and shortened life-span. Concern and empathy were expressed towards people experiencing these effects.

*“And a few…there are quite a few clients that really want to come off antipsychotic drugs*, *such as Olanzapine as a result of the weight gain…because obviously we all know that’s one of the adverse effects of Olanzapine which a lot of clients–even though it might work for them*, *but knocks out their self-confidence and things like that*.*”* P31, Mental Health Nurse, Group 6.

*“I completely get that X and other people’s points that are we unnecessarily giving people obesogenic*, *diabetogenic*, *life-shortening medication when they don’t need it…”* P33, Consultant Psychiatrist, Group 7.

Concerns were also frequently expressed by psychiatrists and a social worker about an over-reliance on antipsychotics in services. These included the use of polypharmacy and rapid dose escalation in order to quickly stabilise and discharge service users from acute services.

#### 1.2 Long-term treatment with antipsychotics is the best option

A small number of psychiatrists (who participated in different focus groups) expressed stronger views that, when weighing up the benefits and drawbacks, antipsychotic medication was unquestionably the best treatment for service users and should be taken long-term. These participants were often sceptical, particularly of antipsychotic discontinuation and cited research showing greater risks of relapse following discontinuation, and their experiences of service-users relapsing after stopping antipsychotics, both with and without their support. The view that antipsychotics protect brain functioning was also expressed.

*“…that’s my starting-point; that these drugs* *work* *and we go to great lengths to persuade patients to keep on* *taking them* *despite side-effects*, *and possible risks because we think that that’s in their best interests of staying well and having a chance of recovery*.*”* P19, Consultant Psychiatrist, Group 4 (underlined words: emphasis).

*“The medication*, *I see it also as a sort of preventative thing for the future and it has been shown that essentially if you keep people on these medications it not only prevents them relapsing but it also protects their brains as it were*, *and protects them for their…improves their future functioning”* P18, Consultant Psychiatrist, Group 4.

### 2. Reduction and discontinuation of antipsychotics in practice

#### 2.1 Reduction as desirable but risky

Given general awareness of adverse effects and concerns from some about overreliance on medication, all psychiatrists described the importance of reducing antipsychotics in some circumstances, and many described experiences of reduction in their clinical practice. Some referred to the idea of trying to attain a ‘minimally effective dose’, with the aim to prevent relapse whilst maximising quality of life.

*“…it’s always the best principle to be on the lowest necessary dose*, *because of side-effect problems*.*”* P20, Consultant Psychiatrist, Group 5.

*“…the guideline that we follow is that with all first-episode psychosis patients usually–I think it’s quite clearly defined–if their episode has lasted less than three months it’s quite easy to make a decision and say we stop their antipsychotic within one to two years after they recover*.*”* P16, Consultant Psychiatrist, Group 3.

While some positive experiences of reduction were reported by both nurses and psychiatrists (often those working in early intervention where reduction and discontinuation are supported by clearer guidelines), the more common experience seemed to be that reduction often resulted in relapse and other adverse outcomes. Practitioners from across the range of professional backgrounds in our sample subscribed to a view that relapse was a likely, or even inevitable, consequence of antipsychotic reduction or discontinuation.

Int: “…So we’d like to start by asking about your experiences of reducing and discontinuing antipsychotics in your own practice.

*P21*: *Relapse—sorry to say*.*”* Consultant Psychiatrist, Group 5.

Accordingly, reduction was approached with caution, due to the risks it was seen to carry. Stability through maintenance treatment was often favoured over reducing antipsychotics, even if this meant that potential over-medication was not tackled, as described in this exchange between two psychiatrists (Group 5).

P23: “I think there’s a general sense of saying it’s been stable for so long, let’s just leave it that way. You just don’t wanna, you know, mess up with somebody who has been well for 15 years and maybe I’ll reduce it.”

P21: “Yeah, because who knows what might happen. But actually they could be on twice as much medication as they need so…”

P23: “Yeah, yeah exactly yeah.”

#### 2.2 Discontinuation as too risky

Given this cautious approach to antipsychotic reduction, stopping these drugs, was not generally considered. This position was described by practitioners working in Community Recovery Services, and some from Early Intervention Services, and was informed by clinicians’ personal experiences of patients who had relapsed following discontinuation. Descriptions of adverse events after both discontinuation that was supported by services/practitioners and unsupported, were more frequent than those after reduction, and included relapse, hospitalisation, criminal offences, drug abuse, and loss of housing, relationships and jobs. Consequently, requests by service-users to stop antipsychotics were described as being more likely to be discouraged than requests to reduce.

*“……unfortunately what we do see in people who against our advice stop their medicines is that they get into a terrible lot of trouble before they get back into the service; you know*, *they lose their relationships*, *they lose their jobs*, *they lose their housing*, *get involved in drug misuse*, *criminality* …*”* P19, Consultant Psychiatrist, Group 4.

In some cases participants felt professionally accountable for these events. One mental health nurse (P2) commented “*…when we want to do that* [stop antipsychotics] *we have to be very cautious…because the result is coming back on the Trust*.*”* There was also the perception of increased treatment resistance following a relapse, with experiences of service users going back onto a higher dose of antipsychotics than they were taking prior to the relapse. Some participants felt that mental health professionals needed to have prior experience of managing relapses and adverse events in order to judge the advisability of antipsychotic reduction or discontinuation.

*“…from my* *own* *personal experience I’ve got to say everyone that I’ve tried to stop*, *all relapsed and* *all* *become unwell*.*”* P34, Consultant Psychiatrist, Group 7.

*“A lot of the care coordinators in the team–especially the newer ones because of the recovery agenda–usually…a lot of them are anti-medication and they’re not*, *you know*, *especially new care coordinators*, *they’ve not seen risk before*, *they’ve not experienced SUIs* [Serious Untoward Incident] *and so unless you've gone through a couple of incidents you don’t know what can happen*, *how close monitoring is required and so on*.*”* P16, Consultant Psychiatrist, Group 3.

### 3. Barriers to reducing antipsychotics

Alongside experiences of adverse events meaning that reduction was approached with caution, attempts to reduce antipsychotics were often described by participants as hampered by significant organisational and knowledge barriers.

#### 3.1 Organisational barriers

**Lack of resources**

Some participants felt that services did not have enough resources to support the reduction process, making maintenance treatment more likely. They discussed a lack of access to and funding for alternative treatments, such as psychological therapies, not enough time to see service users more often when they were in the process of reducing, and not enough resources to support people who may relapse following a reduction.

*“And I might worry slightly about putting extra pressure and demand on the service if we are gonna have more people staying around*, *because they’re relapsing*.*”* P20, Consultant Psychiatrist, Group 5.

“*…That is the thing that I need to know is that…it’s not that the client* [who is reducing] *would be left hanging there without having any resource*, *any back-up*. *Because you try to get an appointment with the consultant it’s difficult enough as it is*. *For them to now come on to this* [reduction study] *even though the consultant might say yeah*, *okay*, *fair enough and then you try to get an appointment and then you hit a block wall…”* P6, Mental Health Nurse, Group 1.

Several groups also discussed how resource cuts over recent years in the UK service context mean there is increasing pressure to rapidly discharge people to primary care once they are stable. This may lead to overmedication, with antipsychotics increased to high doses in order to quickly stabilise and discharge service users. Once discharged, participants described how GPs (primary care physicians) generally do not adjust antipsychotics as they may not feel they have the knowledge, expertise, or clinical support to do this. Antipsychotic discontinuation or even reduction are therefore unlikely to be offered or considered after discharge from secondary care, meaning that many people remain, perhaps unnecessarily, on high doses on a long-term basis. Relatedly, practitioners discussed how rapid discharge may also mean that service users are not in contact with secondary mental health services for long enough to optimise medication regimes, or to attempt to find the ‘minimum effective dose’ of antipsychotics. Frustration was expressed over this issue.

*“…people get put on high levels of medication often they are put on polypharmacy*, *and then as soon as they are not acute enough to need the home treatment team they are discharged*, *often straight back to the GP*, *so there is never the opportunity for stability and there is never the opportunity for de-escalation*, *and the GPs very reasonably do not reduce medication because they do not want to particularly when it is antipsychotic polypharmacy…I think seven or eight years ago…there was much more ability to continue to see people when they were not acutely unwell and to review things and to talk about reductions and plus/minus discontinuation*, *and actually to work out what was the minimum effective dose*.*”* P9, Consultant Psychiatrist, Group 2.

**Poor continuity of care**

Psychiatrists in particular described the difficulty of reducing antipsychotics within a system in which service users frequently move between services at different stages of their illness. This was described as problematic when little or insufficient clinical information about medication decisions was transferred between services. This poor continuity of care may mean that psychiatrists may not have full understanding of the reasons behind medication prescriptions, and/or may not have the time or knowledge of their patient to reduce effectively. Even if a reduction were started, the service user may not be with the psychiatrist long enough to implement the reduction completely.

*“I think also when people come out* [of hospital], *there seems to be this thing of*, *you know people will come out on sort of two antipsychotics and you don’t know actually*, *you can’t really work out were you changing them over*, *was that the idea*? *Or I’m not really sure why this has happened*. *It’s not always clear or…you know*?*”* P21, Consultant Psychiatrist, Group 5.

*“…the problem however is that if it’s* [reduction with the aim to discontinue] *not followed up by one doctor and they get seen by* *several doctors*, *then if you start it* [reduction], *sometimes that’s not followed* *through**…”* P22, Consultant Psychiatrist, Group 5.

#### 3.2 Knowledge barriers

**More guidance and evidence needed to support antipsychotic reduction and discontinuation**

There are currently no professional guidelines on how to reduce and discontinue antipsychotics, and this was highlighted across a number of groups. Participants also felt there was not enough evidence about the outcomes of antipsychotic reduction, and whether it is achievable, safe and worthwhile.

*“we don't know*, *actually whether it is better to reduce or not*, *and therefore whilst there might be a general idea that reducing is a good idea*, *without the evidence it is not unethical to continue at*, *what you anticipate as being a stable dose because we do not actually know that that’s worse*.*”* P9, Psychiatrist, group 2.

With no guidance on how to implement reduction when it was attempted, some psychiatrists spoke about relying on intuition or habit to guide reduction:

*“I would say I*, *I would usually go a bit more slowly 'cos I’d usually say*, *do a reduction maybe every three months*. *But I don’t really have any particular evidence for that; I’ve always just thought three months seems a good length of time to see if there’s going to be a relapse before you reduce it further*.*”* P21, Psychiatrist, Group 5.

**Uncertainty around who may be suitable for reduction**

Psychiatrists also felt that they would like to be able to identify which service users might do better or worse in attempting to reduce antipsychotic dosages. Similarly, some felt that discontinuation was difficult to attempt as there was currently insufficient evidence to predict who might be able to stop antipsychotics successfully. This uncertainty about which service users might be suitable for reduction or discontinuation was highlighted in the wide variety of suggestions regarding people who might benefit from this. These covered a range of factors including illness course and severity: those experiencing a first episode of psychosis, whose mental health appeared stable, were judged to have high levels of insight, were considered low risk, or had uncertain diagnoses; medication usage: people who reported unacceptable side-effects, were taking high doses, or appeared to show antipsychotic treatment resistance; and social circumstances: those thought to have good support networks.

*“But we have patients who are first-episode psychosis which has lasted eight months or a couple of years and they have family histories*, *they’re using cannabis and it’s a bit more difficult decision*. *Of the clear-cut category at least 30% of them recover completely and they don’t require anti-psychotics in the future*. *And as (X) was saying how do you identify that group who don’t require*? *So if we had some research project which would look at that I think that would really help*, *and help the patients as well …”* P16, Psychiatrist, Group 3.

### 4. Communication and negotiation

The importance of open communication between clinicians and service users, particularly in regard to medications, was stressed in all focus groups. Clinicians described how treatment decisions around reduction or discontinuation were made through a process of negotiation between the service user and clinician, often after the service user had made a request to reduce. Related to the views described earlier about long-term antipsychotic use and risks associated with reduction and discontinuation, some psychiatrists reported that they never suggest reduction or discontinuation of antipsychotics, and that this was only ever raised by service users. The importance of respecting and engaging with these requests to reduce was acknowledged by most participants. Some emphasised how they engaged with such requests. Some participants articulated a process of weighing up potential costs and benefits. In supporting requests to reduce medication, they described being motivated by a desire to sustain their relationship with the service user, or to avoid disengagement from services, and/or the risks of unsupported discontinuation.

*“I think so um*, *some people are susceptible to quite marked side-effects*, *especially weight-gain and you know my thinking is it’s better for somebody to take a smaller dose rather than take a higher dose and*, *and stop it–not take it–because of the side-effects*. [Agreement from other group members] *So sometimes it’s worth considering is it better to have a smaller dose and that person will better* [several agree] *adhere to the medication rather than having a too high*, *or a high dose and the side-effects will just like make them stop*, *you know*?*”* P24, Clinical Team Manager, Group 5.

*“I think it’s good for your relationship with people as well*. *If you just sit there going no*, *no*, *no*, *NO*, *NO*, *no [h] we’re not reducing*, *no[h]*. *You know*, *if you actually*, *people feel that you know you’ve said to them*, *you said well look*, *we probably can’t stop this*, *but let’s have a look–let’s try a small reduction and see what happens*, *if that goes okay then we can*, *you know*, *and if you can feel you’re working with them*, *it improves the relationship as well*.*”* P21, Psychiatrist, Group 5.

A small number of psychiatrists felt that their professional knowledge gave them greater decision-making authority than the service user. These psychiatrists described adopting a more paternalistic stance, and in some cases, using a range of strategies to discourage requests to reduce or stop taking antipsychotics. These included relaying the statistical probability of relapse after discontinuation, and drawing parallels between psychosis and chronic physical health conditions such as diabetes.

*“But that’s the group of people I think who*, *who are pressing* [to reduce]*…well maybe I’m wrong 'cos that’s all I see*, *but you know*, *they’re the ones that haven’t yet learnt their lesson of if they stop their medication they relapse”* P17 Psychiatrist, Group 4.

*“we go to great lengths to persuade patients to keep on taking them* [antipsychotics] *despite side-effects*, *and possible risks because we think that that’s in their best interests of staying well and having a chance of recovery…”* P19, Psychiatrist, Group 4.

“*…So I start talking to them about diabetes*, *COPD* [Chronic Obstructive Pulmonary Disease] *and epilepsy and said if you had epilepsy we wouldn’t be stopping your medication*. *You would accept that psychosis is a long-term condition like COPD and diabetes and epilepsy and actually you can’t stop your medication*.*”* P34, Consultant Psychiatrist, Group 7.

Across all focus groups, relationships with families and carers were acknowledged as often providing vital information or support during the reduction process. For many clinicians, families were crucial in spotting early signs of relapse, and were often tasked with managing any initial return of symptoms. However, because of the caring role that families took, participants felt that some families were apprehensive about antipsychotic reduction due to fear of relapse. This could cause conflict if the family and service user had different views on treatment, and clinicians were tasked with managing families’ anxieties.

*“Yeah*, *and they* [the family] *were quite adamant that they didn’t want this to happen* [reduction], *that*, *you know*, *they were even mentioning things*: *oh*, *you're gonna have to section her and*, *you know*, *that kind of line*.*”* P27, Mental Health Nurse, Group 6.

## Discussion

For people with recurrent psychosis, long-term treatment with antipsychotics is standard practice, supported by guidelines [5,6]. However, there are concerns about the consequences of the long-term use of antipsychotics [8,9] and many service users experience unpleasant effects from these drugs and would like to reduce or stop taking them [18,19]. We conducted a focus group study to explore whether and to what extent mental health professionals working in statutory community mental health services in the UK consider and apply antipsychotic reduction or discontinuation, and their attitudes towards this.

Our participants acknowledged the side effect burden of antipsychotics, with some criticising a perceived over-reliance on medication in services. Some felt that antipsychotic maintenance treatment was unquestionably the best option and were often sceptical of reducing and particularly discontinuing antipsychotics, feeling the goal of clinical practice being to ensure adherence. Most participants described the importance of reducing antipsychotics to the lowest effective dose, although complete discontinuation was not generally considered. Prior experiences of adverse events after reduction and discontinuation meant that this was approached with caution, with stability through maintenance treatment often favoured over both reduction and discontinuation. Clinicians considered that organisational factors within mental health services, and knowledge limitations created barriers to reducing antipsychotics. Therefore without appropriate guidelines and evidence, clinicians may be reluctant to implement antipsychotic reduction or discontinuation strategies. They described requests to reduce often coming from the service user first, and actively discouraging discontinuation.

### Clinical and research implications

In the current study, antipsychotic reduction was perceived to be hindered by significant organisational barriers including lack of resources, drive to discharge service users to primary care, and poor continuity of care. These systemic barriers are concerning as they may be hindering good practice. For example, pressure to discharge means that service users may be stabilised quickly through high dose antipsychotics and then discharged to GPs (primary care physicians), who are unlikely to alter this medication [38–40]. This situation reduces patient choice and may result in the long-term use of potentially unnecessarily high doses. A lack of resources, such as clinician time for the additional monitoring and support that safe reduction may require, and limited access to alternative psychosocial treatments means that achieving patient stability through medication is crucial for services to operate effectively. This may mean that maintenance treatment is favoured over reduction for organisational rather than clinical reasons, and that service users may miss out on opportunities to minimise the adverse effects of long-term treatment, or to manage their illness in other ways.

Despite advocating antipsychotic reduction to the minimum effective dose as good clinical practice, clinicians were often reluctant to reduce, meaning that, in line with prior research [30], initiation of reduction processes were often driven by requests from service users. However, research shows that service users are highly aware of unequal power dynamics in medical consultations [25,40,41], and may be reluctant to ask for antipsychotic reduction even when they want this, preferring the suggestion to come from the psychiatrist first [19]. They may *expect* clinicians to make decisions for them, and be afraid to question the authority of the doctor in the desire to be a “good” patient [41]. These dynamic processes in the medical encounter may go some way to explaining why service users perceive a lack of choice about mental health treatments [19,42,43]. Reluctance of professionals to initiate or consider the possibility of antipsychotic reduction or discontinuation is likely to further deter service users from meaningful participation in decisions about their treatment. Service users may become or remain passive or compliant partners in the decision-making process, unable to explore the choices they would prefer in a meaningful way. This situation may also reinforce chronicity and hinder recovery by reducing service-users’ options for managing their mental health problems [26,44]. Although individual clinical encounters take place in the context of a general structural framework of power imbalances within mental health services, encouraging service users to take a more active role in consultations both helps individuals to feel more confident in putting forward their preferences, and starts to address the wider culture that impedes this. Factors that could help to enhance power in individual consultations include a reduction in coercive practices (discussed below), offering service users both drug and non-drug treatment choices such as psychosocial treatments [45], and having frank discussions about the pros and cons of these treatments and expected outcomes [46].

Clinicians’ reluctance to reduce antipsychotics appeared to stem from risk aversion or sometimes scepticism of the feasibility of reduction. Risk aversion stemmed from prior experiences of often serious adverse events and relapse, following reduction or discontinuation. These included suicide, criminal offences, loss of housing, relationships, and jobs. Clinicians’ feared they would be held professionally accountable for such outcomes, especially given the lack of official guidance for antipsychotic reduction. These concerns about risk are in line with clinician attitudes reported in other studies of antipsychotic reduction [31,32]. A small number of psychiatrists were more sceptical of the feasibility of reduction, adopting a more traditional paternalistic stance and believing there was never good justification to try and discontinue antipsychotics and reporting a range of strategies, including relaying statistics, to persuade people to stay on antipsychotics.

These sceptical attitudes may relate to issues of risk aversion previously discussed, but they may also stem from the widely accepted medical model of psychosis itself—that suggests that antipsychotics correct a ‘chemical imbalance’ and are therefore necessary for recovery [47]. Even though this model is the subject of considerable debate [48,49], for many psychiatrists it is regarded as established fact. Previous research has found that over-reliance on a scientific model of treatment along with potentially coercive practices, can undermine the trust, choice and power service-users have in their mental health treatment [46]. It has been suggested that coercion in mental health practice could be seen as a form of epistemic injustice, where clinicians do not see the service-users’ knowledge and decision making ability as legitimate due to the beliefs they hold about their diagnosis [50]. For example people with schizophrenia may be viewed as out of touch with reality, diminishing the legitimacy of their knowledge and requests, and increasing the possibility of more coercive or controlling treatment [50,51]. These attitudes could form the context of individual clinical decisions. In our data, for example, service user preferences often appeared to be trumped by scepticism and risk aversion which favoured the stability associated with maintenance treatment. Against this background, increasing the involvement of service users in treatment decisions, as recommended by national guidelines [5], is challenging, and is likely to require changes in wider cultural attitudes within services. Genuine shared-decision making in this context will require an openness to discussing antipsychotic reduction [25–28]. One way of enabling this would be through the development of formal guidelines for reduction and discontinuation, to help psychiatrists feel safer and more supported when reducing antipsychotics.

It was clear from our focus groups that mental health professionals, particularly psychiatrists, felt they needed more evidence-based guidance regarding whether, how and with whom antipsychotics can be reduced or stopped safely. Research to date has failed to provide any consistent predictors of successful reduction or discontinuation [52,53]. This absence of evidence and guidelines may discourage clinicians from considering reduction of antipsychotic treatment as standard, as our participants’ descriptions of treatment decisions and discussions with service users suggest.

Current guidelines recommend family involvement as best practice, however service-user—family/carers views on antipsychotic reduction were often described as conflicting, hindering the implementation of this recommendation and also shared decision making. Families and carers have an important role in supporting service-users but were described as sometimes being concerned or resistant to antipsychotic reduction, with psychiatrists and clinical teams having to negotiate reduction and manage the families concerns. This may be due to the responsibility family and carers feel for antipsychotic compliance, leading them to take a more controlling role in medication decisions [42]. The identified organisational barriers could be linked to these issues. Under-resourced services may mean more responsibility is placed on family and carers, with clinicians finding it difficult to provide reassurance that the appropriate increased support will be available when reducing. Rapid discharge from secondary services and lack of continuity of care may make it difficult to build trusting relationships and good communication with families and carers which would help to allay concerns. Clinical guidelines should provide information on how to manage conflicting demands from patients and their families or carers and how best to address the latter’s legitimate concerns.

### Strengths and limitations

To our knowledge this is the first study to specifically investigate the views of a range of mental health professionals working in secondary care on reducing and stopping antipsychotics. Psychiatrists, psychiatric nurses and other professionals from a range of mental health services that support people with both short-term and longer-term mental health problems were included, maximising our ability to explore a diversity of views and experiences from different perspectives.

Limitations include the sample consisting primarily of consultant psychiatrists and nurses, and that several focus groups were made up of clinicians who worked on teams together, or knew each other through peer networks. While this may have put participants at ease, they may also have been reluctant to divulge service-related concerns or differences of opinion in front of colleagues or managers. Participants’ were aware of the facilitators’ positionality, i.e. their involvement in the RADAR antipsychotic reduction study. Although no further details regarding researchers’ views on antipsychotics were disclosed, we recognise that this awareness and potential interpretation that facilitators were supportive of antipsychotic reduction could have influenced some views. Participants may have self-censored critical comments, or conversely emphasised contrary views more strongly. We collected data in areas of London with rapid demographic expansion and consequent high pressure on psychiatric beds. This may reduce the applicability of findings to other areas.

### Conclusion

While long-term antipsychotic treatment may successfully reduce psychosis symptoms and prevent relapse for some [2–4], it is known to be associated with significant health consequences, and can be subjectively and objectively impairing [8]. Relapse following reduction or discontinuation is not inevitable [23], and some service users reasonably wish to reduce their side-effect burden, or consider options other than continuous maintenance treatment. However, when service users stop antipsychotics covertly and abruptly (which may be more likely if support or options to reduce are not offered) their risk of relapse and negative or coercive service experiences may increase. More research is needed into the outcomes of antipsychotic reduction and discontinuation that is clinically supported and conducted systematically, gradually and responsively. This could inform evidence-based guidelines on reduction and discontinuation processes that are currently not available. Our findings suggest that practitioners would greatly value such guidelines, which could enable them to feel more confident in responding to service users’ requests or preferences to alter or reduce medication. In turn, this would enable service users to be more involved in managing their mental health. In addition, it is important to consider how organisational or systemic factors could enable rather than impede clinicians in these processes. Further support for engaging patients meaningfully and constructively in decision making would be useful, and financial constraints that encourage the prioritisation of short-term stability to enable rapid discharge, with long term high-dose antipsychotic prescribing need to be debated.

## Supporting information

S1 Appendix Topic guides. (DOCX)
