# Supplementary material for: Mental health professionals’ views and experiences of antipsychotic reduction and discontinuation
Source: PLoS One. 2019 Jun 20;14(6):e0218711. doi: 10.1371/journal.pone.0218711 (PMC6586401; doi:10.1371/journal.pone.0218711)
Supplement: S1 Appendix — (DOCX) [file pone.0218711.s001.docx]

**S1 Appendix: topic guides**

Begin with an introduction about why you are here and a brief summary of the RADAR (Research into Antipsychotic Discontinuation and Reduction) study. Let people know what you are going to do with the data. Reassure people that you are not coming to assess their performance – you are looking for advice and guidance on how we can make the RADAR programme better.

Introduction and orientation to topic – *for both psychiatrists and senior members of the clinical team.*

Take tape recorder out, distribute handouts (RADAR summary) and ask participants to fill out demographics.

*“I would like to record our conversation today – is that OK? That’s so I can really listen to what you say”* [If refuses tape recorder, ask for permission to take notes].

*“I am here today to talk to you about the RADAR study which stands for Research into Antipsychotic Discontinuation and Reduction. The main aims of the RADAR programme are to see whether guided reduction and discontinuation of antipsychotics can be achieved safely and improve functioning. The main part of this study is a randomised controlled trial comparing an active antipsychotic reduction/discontinuation arm with a treatment as usual arm. In the active arm, the patient’s psychiatrist will work with the patient to reduce and potentially discontinue their antipsychotic medication.”*

*“As we talk today there are no right or wrong answers at all – we are interested in your views on how we propose to implement the antipsychotic reduction strategy, and the trial as a whole, based on any relevant experience you’ve had. Once I leave here today our conversation will be typed up.”*

*“At no time will your name or personal details be attached to the written version of our conversation or any materials that we publish. The information you give us is treated in confidence.”*

*“Members of the research team will need to listen to the recording of the conversation and read the written version of our conversation. The research team will use the information you give us today to think about how we can develop your ideas, so it is very helpful that you have agreed to talk to us today”*

**Topic guide – *psychiatrists***

Section 1. Experiences and views of reducing / discontinuing antipsychotics (spend ~10 minutes).

*"We'd like to start by asking you about your experiences of reducing and discontinuing antipsychotics"*

Prompts:

- Do you use a reduction/discontinuation strategy already?
  - In what circumstances and with what sort of patients would you use such a strategy?
  - What do you think are the advantages, disadvantages and risks of reducing and potentially discontinuing antipsychotic medication?
  - In your experience of using a reduction/discontinuation strategy:
    - At what rate and over what period do you typically reduce medication?
    - Do you ever stop antipsychotic medication completely? If so in what circumstances?
    - What methods of reducing/discontinuing have worked or haven’t worked well and why?

Section 2. Their views of the specific processes proposed for RADAR (20-30 mins)

Distribute handouts (reduction protocol)

*“Now that you have read about the study, what we would like you to particularly look at is the reduction schedules. [indicate to the current example reduction schedules]. We would like to get your views on it generally but we are particularly interested in your views on a few specific things.”*

- *Thinking about what we have just discussed, about your general views on antipsychotic reduction and discontinuation, what do you think of the draft antipsychotic reduction protocols for RADAR?*

*Direction: WRITE THESE FOUR QUESTIONS BELOW ON THE WHITE BOARD*

1. *What do you think of the proposed length of the reduction period?*

*2. What do you think about proposed size of the dose reductions in the example schedules?*

*3.What do you think of the proposed frequency of reviews (two monthly) for patients in the intervention group?*

- *What do you think will facilitate or support a reduction /discontinuation strategy?*
- *What do you think will be the barriers to reducing/discontinuing antipsychotic medication?*
  - *Would you personally have any concerns about participating in the RADAR study?*

*4.What would you think about delivering both the reduction and maintenance interventions?*

- *I.e. if two of your patients were randomised to these different interventions*

Prompts:

- - What suggestions for overcoming these concerns do you have?
  - Are there any patients that you think would be particularly suitable or unsuitable for these antipsychotic reduction strategies?

Section 3. Relapse: (5 mins)

- *One of the outcomes of RADAR will be looking at relapse rates. We want to make sure we are defining and measuring relapse in the best way possible. We were wondering how you would define relapse?*

Prompts:

- - What do you think a ‘relapse’ in schizophrenia or psychosis consists of?
  - How do you think it is best to define relapse?

Section 4. Sources of support: (5-10 mins)

- *We want to give all participants in the trial general advice on staying well during the process of reduction what would you consider to be important in order to support them?*

Prompts

- - Do you work with any patients with psychosis who are unmedicated or on low doses of their antipsychotics - what sources of support do they find helpful?
- Conclusion and debrief:
- *Thinking about everything we have discussed today what do you think is most important for us to consider when designing the reduction/discontinuation strategy?*

***---------------------------------------------------------------------------------------------------------------------***

**Topic guide – *members of the clinical team***

Section 1. Experiences and views of reducing / discontinuing antipsychotics (spend ~10 minutes).

- *We'd like to start by asking you about your experiences of working with patients who are reducing or discontinuing their antipsychotics*

Prompts:

- In what circumstances and with what sort of patients has a reduction/discontinuation strategy worked well?
- In what circumstances and with what sorts of patients has a reduction/discontinuation strategy not worked well? Probe perceived reasons if necessary.

Section 2. General views on the RADAR study (20 mins)

- *Now that you have read about the study [indicate handouts] would you have any concerns about patients taking part?*
- *What suggestions for overcoming these concerns do you have?*
- *Are there any patients that you think would be suitable/unsuitable for the reduction/discontinuation strategy?*
- *What do you think will facilitate/help patients reduce/discontinue their antipsychotic medication?*
- *What do you think will be the barriers for patients to reduce/discontinue their antipsychotic medication?*

Section 3. Relapse (5 mins)

- *One of the outcomes of RADAR will be looking at relapse rates, we want to make sure we are defining and measuring relapse in the best way possible, we were wondering how you would define relapse?*

Prompts:

- What do you think a ‘relapse’ in schizophrenia or psychosis consists of?
- How do you think it is best to define relapse?

Section 4. Sources of support (10 mins)

- *We want to give all participants in the trial general advice on staying well during the process of reduction. What would you consider to be important in order to support them?*

Prompts

- Do you work with any patients with psychosis who are unmedicated or on low doses of their antipsychotics - what sources of support do they find helpful?

Conclusion and debrief: (5 mins)

- *Thinking about everything we have discussed today what do you think is most important for us to consider when planning the RADAR study?*
